# A mixture model approach to sample size estimation in two-sample comparative microarray experiments

**DOI:** 10.1186/1471-2105-9-117

**Published:** 2008-02-25

**Authors:** Tommy S Jørstad, Herman Midelfart, Atle M Bones

**Affiliations:** 1 Department of Biology, Norwegian University of Science and Technology, Høgskoleringen 5, NO-7491 Trondheim, Norway

## Abstract

**Background:**

Choosing the appropriate sample size is an important step in the design of a microarray experiment, and recently methods have been proposed that estimate sample sizes for control of the False Discovery Rate (FDR). Many of these methods require knowledge of the distribution of effect sizes among the differentially expressed genes. If this distribution can be determined then accurate sample size requirements can be calculated.

**Results:**

We present a mixture model approach to estimating the distribution of effect sizes in data from two-sample comparative studies. Specifically, we present a novel, closed form, algorithm for estimating the noncentrality parameters in the test statistic distributions of differentially expressed genes. We then show how our model can be used to estimate sample sizes that control the FDR together with other statistical measures like average power or the false nondiscovery rate. Method performance is evaluated through a comparison with existing methods for sample size estimation, and is found to be very good.

**Conclusion:**

A novel method for estimating the appropriate sample size for a two-sample comparative microarray study is presented. The method is shown to perform very well when compared to existing methods.

## Background

One of the most frequently used experimental setups for microarrays is the two-sample comparative study, i.e. a study that compares expression levels in samples from two different experimental conditions. In the case of replicated two-sample comparisons statistical tests may be used to assess the significance of the measured differential expression. A natural test statistic for doing so is the t-statistic (see e.g. [1]), which will be our focus here. In the context of two-sample comparisons it is also convenient to introduce the concept of 'effect size'. In this paper effect size is taken to mean: the difference between two conditions in a gene's mean expression level, divided by the common standard deviation of the expression level measurements.

In an ordinary microarray experiment thousands of genes are measured simultaneously. Performing a statistical test for each gene leads to a multiple hypothesis testing problem, and a strategy is thus needed to control the number of false positives among the tests. A successful approach to this has been to control the false discovery rate (FDR) [2], or FDR-variations like the positive false discovery rate (pFDR) [3].

To obtain the wanted results from an experiment it is important that an appropriate sample size, i.e. number of biological replicates, is used. A goal can, for example, be set in terms of a specified FDR and average power, and a sample size chosen so that the goal may be achieved [4].

In the last few years many methods have been suggested that can help estimate the needed sample size. Some early approaches [5,6] relied on simulation to see the effect of sample size on the FDR. Later work established explicit relationships between sample size and FDR. A common feature of the more recent methods is that they require knowledge of the distribution of effect sizes in the experiment to be run. In lack of this distribution there are two alternatives. The first alternative is simply specifying the distribution to be used. The choice may correspond to specific patterns of differential expression that one finds interesting, or it can be based on prior knowledge of how effect sizes are distributed. Many of the available methods discuss sample size estimates for specified distributions [7-11]. The second alternative is estimating the needed distribution from a pilot data set. Ferreira and Zwinderman [12] discuss one such approach. Assuming that the probability density functions for the test statistics are symmetric and belonging to a location family, they obtain the wanted distribution using a deconvolution estimator. One should note that, for the sample sizes often used in a microarray experiment, the noncentral density functions for t-statistics depart from these assumptions. Hu *et al*. [13] and Pounds and Cheng [14] discuss two different approaches. Both methods recognize that test statistics for differentially regulated genes are noncentrally distributed, and aim to estimate the corresponding noncentrality parameters. From the noncentrality parameters, effect sizes can be found. Hu *et al*. consider, as we do, t-statistics and estimate the noncentrality parameters by fitting a 3-component mixture model to the observed statistics of pilot data. Pounds and Cheng consider F-statistics and estimate a noncentrality parameter for each observation. They then rescale the estimates according to a Bayesian q-value interpretation [15]. A last approach that needs mention is that of Pawitan *et al*. [16], which fits a mixture model to observed t-statistics using a likelihood-based criterion. The approach is not explored as a sample size estimation method in the paper by Pawitan *et al*., but it can be put to this use.

In this article we introduce a mixture model approach to estimating the underlying distribution of noncentrality parameters, and thus also effect sizes, for t-statistics observed in pilot data. The number of mixture components used is not restricted, and we present a novel, closed form, algorithm for estimating the model parameters. We then demonstrate how this model can be used for sample size estimation. By examining the relationships between FDR and average power, and between FDR and the false nondiscovery rate (FNR), we are able to choose sample sizes that control these measures in pairs. To validate our model and sample size estimates, we test its performance on simulated data. We include the estimates made by the methods of Hu *et al*., Pounds and Cheng and Pawitan *et al*.

## Results and Discussion

### Notation, assumptions and test statistics

Throughout this text *t*_*ν *_(*λ*) represents the probability density function (pdf) of a t-distributed random variable with *ν *degrees of freedom and noncentrality parameter *λ*. A central t pdf, *t*_*ν *_(0), can also be written *t*_*ν*_. A *t*_*ν *_(*λ*) evaluated at *x *is written *t*_*ν *_(*x*; *λ*).

Assume gene expression measurements can be made in a pilot study. For a particular gene we denote the *n*_1 _measurements from condition 1 and the *n*_2 _from condition 2 by *X*_1*i*_, (*i *= 1, ..., *n*_1_) and *X*_2*j*_, (*j *= 1, ..., *n*_2_). Let (*μ*_1_, $\sigma_{1}^{2}$) and (*μ*_2_, $\sigma_{2}^{2}$) be expectation and variance for each *X*_1*i *_and *X*_2*j*_, respectively. For simplicity we focus in this paper on the case where $\sigma_{1}^{2}=\sigma_{2}^{2}=\sigma^{2}$. As is common in microarray data analysis, the *X*_1*i*_s and *X*_2*j*_s are assumed to be normally distributed random variables.

Measured expression levels are often transformed before normality is assumed.

A statistic frequently used to detect differential expression in this setting, is the t-statistic. Two versions of t-statistics can be used, depending on the experimental setup. In the first setup, measurements for each condition are made separately. Inference is based on the two-sample statistic

(1) $$T_{1}=\frac{\sqrt{n_{1}n_{2}{\left(n_{1}+n_{2}\right)}^{-1}}({\bar{X}}_{1}-{\bar{X}}_{2})}{\sqrt{{\left(n_{1}+n_{2}-2\right)}^{-1}[(n_{1}-1)S_{1}^{2}+(n_{2}-1)S_{2}^{2}]}},$$

where ${\bar{X}}_{k}=n_{k}^{-1}\sum_{i=1}^{n_{k}}X_{ki}$ and $S_{k}^{2}={\left(n_{k}-1\right)}^{-1}\sum_{i=1}^{n_{k}}{\left(X_{ki}-{\bar{X}}_{k}\right)}^{2}$. Under the null hypothesis *H*_0 _: *μ*_1 _= *μ*_2_, *T*_1 _has a $t_{n_{1}+n_{2}-2}$ pdf. If, however, *H*_0 _is not true, and there is a difference *μ*_1 _- *μ*_2 _= *ξ*, the pdf of the statistic is a $t_{n_{1}+n_{2}-2}(\xi \sigma^{-1}\sqrt{n_{1}n_{2}{\left(n_{1}+n_{2}\right)}^{-1}}$. This setup includes comparing measurements from single color arrays and two-color array reference designs. In a second kind of experiment, measurements are paired. In the case of, for example, *n *two-color slides that compare the two conditions directly, then *n*_1 _= *n*_2 _= *n*, and the statistic used is

(2) $$T_{2}=\frac{\bar{d}}{\sqrt{S_{d}^{2}/n}},$$

where $\bar{d}=n^{-1}\sum_{i=1}^{n}\left(X_{1i}-X_{2i}\right)$ and $S_{d}^{2}={\left(n-1\right)}^{-1}\sum_{i=1}^{n}{\left(d_{i}-\bar{d}\right)}^{2}$. Now, under *H*_0 _: *μ*_1 _= *μ*_2_, *T*_2 _has as a *t*_*n *- 1 _pdf. If *μ*_1 _- *μ*_2 _= *ξ*, however, the pdf of *T*_2 _is a *t*_*n *- 1 _(*ξ σ*^-1 ^$\sqrt{n}$).

In both experimental setups we note that the pdf of t-statistics for truly unregulated genes is *t*_*ν *_(0). For truly regulated genes the pdf is *t*_*ν *_(*δ*), with *δ *≠ 0 reflecting the gene's level of differential expression. We also note that this *δ *is proportional to the gene's effect size, *ξ*/*σ *. The *δ*s can be considered realizations of some underlying random variable Δ, distributed as *h*(*δ*). Under our assumptions the observed t-scores should thus be modelled as a mixture of *t*_*ν *_(*δ*)-distributions, with the *h*(*δ*) as its mixing distribution. The *h*(*δ*) is not directly observed and must be estimated.

In the following, the t-statistics calculated in an experiment are assumed to be independent. This assumption, and the assumption that $\sigma_{1}^{2}=\sigma_{2}^{2}=\sigma^{2}$, may not hold in the microarray setting. In the Testing section we examine cases where these assumptions are not satisfied to see how results are affected.

### Algorithm for estimating effect sizes

Let *y*_*j*_, *j *= 1, ..., *m *denote observed t-statistics for the *m *genes of an experiment, having ***Y***_*j*_s as corresponding random variables. Let *f*(*y*) be their pdf. Our mixture model can then generally be stated as

*f*(*y*; *h*) = ∫ *t*_*ν *_(*y*; *δ*) *dh*(*δ*),

where *h*(*δ*) is any probability measure, discrete or continuous. To estimate *h*(*δ*) we want to find a probability measure that maximizes the likelihood, *L*(*h*), of our observations, where

$$L(h)=\prod_{i=1}^{m}f(y_{i};h).$$

It has been shown [17] that to solve this maximization problem, when *L*(*h*) is bounded, it is sufficient to consider only discrete probability measures with *m *or fewer points of support. Motivated by this we choose *h*(*δ*) to be a discrete probability measure, and aim to fit a mixture model of the form

(3) $$f(y)=\sum_{i=0}^{g}\pi_{i}t_{\nu }(y;\delta_{i})=\pi_{0}t_{\nu }(y;0)+\sum_{i=1}^{g}\pi_{i}t_{\nu }(y;\delta_{i}).$$

The *h*(*δ*) is thus a distribution where Pr(*δ *= *δ*_*i*_) = *π*_*i*_, (*i *= 0, ..., *g*), and $\sum_{i=0}^{g}\pi_{i}=1$. The second form of *f*(*y*) in (3) is due to knowing that *δ *= 0 for unregulated genes.

We now aim to find the parameters of a model like (3) with a fixed number of components *g *+ 1. It is clear that finding these parameters can be formulated as a missing data problem, which suggests the use of the EM-algorithm [18]. Although this approach has been discussed in earlier work [13,16], a closed form EM-algorithm that solves the problem has not been available until now. The main difficulty with constructing the algorithm is the lack of a closed form expression for the noncentral t pdf. In the remainder of this section we show how the needed algorithm can be obtained.

As is usual with EM, random component-label vectors **Z**_1_, ..., **Z**_*m *_are introduced that define the origin of *Y*_1_, ..., *Y*_*m*_. These have *Z*_*ij *_= (*Z*_*j*_)_*i *_equal to one or zero according to whether or not *Y*_*j *_belongs to the *i*th component. A **Z**_*j *_is distributed according to

$$\mathrm{Pr}(Z_{j}=z_{j})=\pi_{0}^{z_{0j}}\pi_{1}^{z_{1j}}\cdots \pi_{g}^{z_{gj}},$$

where the **z**_*j *_is a realized value of the random **Z**_*j*_.

We proceed by recognizing the fact that a noncentral t pdf of is itself a mixture. The cumulative distribution of a variable distributed according to *t*_*ν *_(*δ*) is (see [19])

$$F_{\nu }(y;\delta )=\frac{1}{2^{\frac{\nu }{2}}\Gamma \left(\frac{\nu }{2}\right)}\int_{0}^{\infty }v^{\nu -1}e^{\frac{-v^{2}}{2}}\frac{1}{\sqrt{2\pi }}\int_{-\infty }^{\frac{yv}{\sqrt{\nu }}}e^{-\frac{1}{2}{\left(s-\delta \right)}^{2}}dsdv.$$

Differentiating *F*_*ν *_(*y*) with respect to *y*, and substituting $v=\sqrt{\nu u}$, yields

(4) $$F_{\nu }(y;\delta )=\int_{0}^{\infty }\frac{u}{\sqrt{2\pi }}e^{-\frac{1}{2}{\left(y-\frac{\delta }{u}\right)}^{2}u^{2}}\frac{\sqrt{\nu }}{2^{\frac{\nu }{2}-1}\Gamma \left(\frac{\nu }{2}\right)}{\left(\sqrt{\nu }u\right)}^{\nu -1}e^{-\frac{\nu u^{2}}{2}}du.$$

This form of the noncentral t pdf can be identified as a mixture of normal $N\left(\frac{\delta }{u},\frac{1}{u^{2}}\right)$ distributions, with a scaled *χ*_*ν *_mixing distribution for the random variable *U*. Based on the characterization in (4) we can introduce a new set of missing data u_*ij*_, (*i *= 0, ..., *g*; *j *= 1, ..., *m*) that are realizations of *U*_*ij*_, and defined so that (*Y*_*j*_|*u*_*ij*_, *z*_*ij *_= 1) follows a $N\left(\frac{\delta }{u_{ij}},\frac{1}{u_{ij}^{2}}\right)$ distribution. Restating the model in this form, as a mixture of mixtures, is a vital step in finding the closed form algorithm.

The *y*_*j*_s augmented by the *z*_*ij*_s and *u*_*ij*_s form the complete-data set. The complete-data log-likelihood may be written

(5) $$\log L_{c}(\pi ,\delta )=\sum_{i=0}^{g}\sum_{j=1}^{m}z_{ij}\log(\pi_{i}f_{c}(y_{j}|u_{ij},z_{ij}=1)g_{c}(u_{ij})),$$

where ***π ***= (*π*_1_, ..., *π*_*g*_), ***δ ***= (*δ*_1_, ..., *δ*_*g*_) and *f*_*c*_(*y*|*u*, *z*) and *g*_*c*_(*u*) are the above-mentioned normal and scaled *χ*_*ν *_distribution, respectively. The E-step of the EM-algorithm requires the expectation of *L*_*c *_in (5) conditional on the data. Combining (4) with (5) we find that we need the expectations

*E*(*Z*_*ij*_|*y*_*j*_) and *E*(*U*_*ij*_|*y*_*j*_, *z*_*ij *_= 1).

Calculating the first expectation is straightforward (see e.g. [20]) and is found to be

(6) $$E(Z_{ij}|y_{j})=\frac{\pi_{i}t_{\nu }(y_{j};\delta_{i})}{\sum_{i=0}^{g}\pi_{i}t_{\nu }(y_{j};\delta_{i})}.$$

Calculating the second expectation is harder, but by using Bayes theorem we find that it can be stated as (now omitting indices for clarity)

(7) $$E(U_{ij}|y_{j},z_{ij}=1)=\int_{0}^{\infty }uf_{c}(u|y,z)du$$

(8) $$=\int_{0}^{\infty }\frac{uf_{c}(y|u,z)g_{c}(u)}{\int_{0}^{\infty }f_{c}(y|u^{\prime },z)g_{c}(u^{\prime })du^{\prime }}du$$

where *f*_*c*_(*u*|*y*, *z*) is the pdf of (*U*_*ij*_|*y*_*j*_, *z*_*ij *_= 1). Note that *g*_*c*_(*u*|*z*) = *g*_*c*_(*u*) since *U*_*ij *_and *Z*_*ij *_are independent.

The integral in (8) must now be evaluated. To do this, we note that the denominator of the integrand is itself an integral and that it does not depend on the integrating variable *u*. In effect, (8) is thus the ratio of two integrals. After a substitution of variables in both integrals, $\left(w=u\sqrt{y^{2}+\nu }\right)$ and $\left(w=u^{\prime }\sqrt{y^{2}+\nu }\right)$, we find that this ratio can be rewritten in terms of *H h*-functions as

(9) $$E(U_{ij}|y_{j},z_{ij}=1)=\frac{\nu +1}{\sqrt{y_{j}^{2}+\nu }}\frac{Hh_{v+1}\left(\frac{-y_{j}\delta_{i}}{\sqrt{y_{j}^{2}+v}}\right)}{Hh_{v}\left(\frac{-y_{j}\delta_{i}}{\sqrt{y_{j}^{2}+v}}\right)},$$

where $Hh_{k}(x)=\int_{0}^{\infty }\frac{w^{k}}{k!}e^{-\frac{1}{2}{\left(w+x\right)}^{2}}$*dw *for an integer *k *≥ 0. The properties of the *H h*_*k*_-functions are discussed in [21]. A particularly nice property is that it satisfies the recurrence relation

(*k *+ 1) *H h*_*k *+ 1 _(*x*) = - *x H h*_*k *_(*x*) + *H h*_*k *- 1 _(*x*).

With easily calculated $Hh_{-1}(x)=e^{-\frac{1}{2}x^{2}}$, $Hh_{0}(x)=\int_{x}^{\infty }e^{-\frac{1}{2}u^{2}}du$, we have a convenient way of computing (9).

The M-step requires maximizing the *L*_*c *_with respect to ***π ***and ***δ***. This is accomplished by simple differentiation and yields maximizers

(10) $${\hat{\pi }}_{i}=\frac{1}{m}\sum_{j=1}^{m}z_{ij}$$

(11) $${\hat{\delta }}_{i}=\frac{\sum_{j=1}^{m}z_{ij}y_{j}u_{ij}}{\sum_{j=1}^{m}z_{ij}}.$$

Equations (6) and (9) – (11) constitute the backbone of the needed closed form EM-algorithm to fit a mixture model like (3) with *g *+ 1 components. Parameter estimates are updated according to the scheme

$$\begin{array}{l} \begin{array}{ccc} i.) & u_{ij}^{\left(k\right)}=E_{{\hat{\pi }}^{\left(k\right)}{\hat{\delta }}^{\left(k\right)}}(U_{ij}|y_{j},z_{ij}=1), & z_{ij}^{\left(k\right)}=E_{{\hat{\pi }}^{\left(k\right)}{\hat{\delta }}^{\left(k\right)}}(Z_{ij}|y_{j}) \end{array}\\ \begin{array}{ccc} ii.) & {\hat{\pi }}_{i}^{\left(k+1\right)}=\frac{1}{m}\sum_{j=1}^{m}z_{ij}^{\left(k\right)}, & {\hat{\delta }}_{i}^{\left(k\right)}=\frac{\sum_{j=1}^{m}z_{ij}^{\left(k\right)}y_{j}u_{ij}^{\left(k\right)}}{\sum_{j=1}^{m}z_{ij}^{\left(k\right)}}. \end{array} \end{array}$$

On convergence, the estimated ***π ***and ***δ ***are used as the parameters of a *h*(*δ*) with *g *+ 1 point masses. As discussed above we fix *δ*_0 _= 0.

An issue that has received much attention is estimating the proportion of true null hypotheses when many hypothesis tests are performed simultaneously (e.g. [3,22,23]). In the microarray context this amounts to estimating the proportion of truly unregulated genes among all genes considered. Referring to (3) we see that this quantity enters our model as *π*_0_. To draw on the extensive work on *π*_0_-estimation, we suggest using a known conservative *π*_0_-estimate to guide the choice of some model parameters. This is discussed below. In our implementation we use the convex decreasing density estimate proposed in [24], but a different estimate may be input.

Assessing the appropriate number of components in a mixture model is a difficult problem that is not completely resolved. An often adopted strategy is using measures like the Akaike Information Criterion (AIC) [25] or the Bayesian Information Criterion (BIC) [26]. We find from simulation that, in our setting, these criteria seem to underestimate the needed number of components (refer to the Testing section for some of the simulation results). This is possibly due to the large proportion of unregulated genes often found in microarray data. With relatively few regulated genes, the gain in likelihood from fitting additional components is, for these criteria, not enough to justify the additional parameters used. In our implementation we use *g *= log_2_((1 - ${\hat{\pi }}_{0}$) *m*), where ${\hat{\pi }}_{0}$ is the above-mentioned estimate. This choice is motivated by experience, but has proven itself adequate in our numerical studies. It also reflects the fact that a single component should provide sufficient explanation for the unregulated genes, while the remaining *g *components explain the regulated ones. A different *g *may be specified by users of the sample size method.

A complication that could arise when fitting the mixture model is that one or more of the ${\left\{\delta_{i}\right\}}_{i=1}^{g}$, could be assigned to *δ *= 0, or very close to it, thus affecting the fit of the central component. To avoid this, we define a small neighbourhood around *δ*_0 _= 0 from which all *g *remaining components are excluded. A *δ*_*i*_, *i *≠ 0, that tries crossing into this neighbourhood while fitting the model, is simply halted at the neighbourhood boundary. The boundary is determined by finding the smallest $\tilde{\delta }$ for which it is possible to tell apart the *t*_*ν *_(0) distribution from a *t*_*ν *_($\tilde{\delta }$) one, based on samples of sizes ${\hat{\pi }}_{0}m$ and (1 - ${\hat{\pi }}_{0}$) *m/g*, respectively. The latter sample size assumes regulated genes to be evenly distributed among their components. The samples are taken as evenly spaced points within the 0.05 and 0.95 quantiles of the two distributions. The criterion used to check if the two samples originated from the same distribution is a two-sample Kolmogorov-Smirnov test with a significance level of 0.05. The rationale behind this criteron is that, for the *g *components associated with regulated genes, we only want to allow those that with reasonable certainty can be distinguished from the central component. Again, the ${\hat{\pi }}_{0}$ used is the estimate discussed above.

Another difficulty related to fitting a mixture model is that the optimal fit is not unique, something which might cause convergence problems. The difficulty is due to the fact that permuting mixture components does not change the likelihood function. In our implementation we do not have any constraints that resolve this problem. We did, however, track the updates of our mixture components in a number of test runs and did not see this problem occur.

In summary, our approach provides estimates, ${\left\{{\hat{\delta }}_{i}\right\}}_{i=0}^{g}$ and ${\left\{{\hat{\pi }}_{i}\right\}}_{i=0}^{g}$, of the noncentrality parameters in the data and a set of weights. Together these quantities make up an estimate of the distribution *h*(*δ*). As seen in the section on test-statistics a *δ *is proportional to the effect size, *ξ*/*σ*. No estimates or assumptions are thus made on the numerical size of the means or variances in the data. We only estimate a set of mean shift to variance ratios.

### Algorithm for estimating sample sizes for FDR control

An important issue in experimental design is estimating the sample size required for the experiment to succeed. We now outline how to choose sample sizes that control FDR together with other measures, and how the model discussed above can be used for this purpose.

Table 1 summarizes the possible outcomes of *m *hypothesis tests. All table elements except *m *are random variables. From Table 1 the much used positive false discovery rate (pFDR) [3] can be defined as E(*R*_0_/*M*_*R*_|*M*_*R *_> 0). In [3] it is also proven that, for a given significance region and under assumed independence for the tests, we have

**Table 1:** Outcomes of *m *hypothesis tests

	**H_0 _accepted**	**H_0 _rejected**	**Total**
**H_0 _true**	*A*_0_	*R*_0_	*m*_0_
**H_0 _false**	*A*_1_	*R*_1_	*m*_1_
**Total**	*M*_*A*_	*M*_*R*_	*m*

(12) pFDR = Pr(H_0 _true|H_0 _rejected),

and that, in terms of p-values *p *and a chosen p-value cutoff *α*, (12) can be rewritten as

(13) $$\text{pFDR}(\alpha )=\frac{\pi_{0}\alpha }{\mathrm{Pr}(p<\alpha )}.$$

Equation (13) is important in sample size estimation as it provides the relationship between pFDR, significance region and sample size. Sample size will determine Pr(*p *<*α*) since the shape of the t-distributions depends on *n*_1_, *n*_2_. (An explanation is provided at the end this section). The application of (13) to sample size estimation was first discussed by Hu *et al*. [13].

Using (13), and a fitted mixture model, one can estimate the sample size that achieves a specified *α *and pFDR. The remaining issue is choosing an appropriate *α *and pFDR. The pFDR is an easily understood measure, and its size can be set directly by users of the sample size estimation method. How to pick *α*, on the other hand, is not as clear. One solution to this is to restate (13) as relationships between *α*, pFDR and other statistical measures. Hu *et al*. present one such relationship. They suggest picking the *α *by specifying the expected number of hypotheses to be rejected, E(*M*_*R*_). Their idea is substituting Pr(*p *<*α*) = E(*M*_*R*_)/*m *in (13) to get

(14) $$\alpha =\frac{\text{pFDR}}{\pi_{0}}\frac{\text{E}(M_{R})}{m}.$$

In words, instead of specifying an *α*, one can specify E(*M*_*R*_). This way of obtaining *α*, however, has a shortcoming. It provides little direct information to the user about the experiment's ability to recognize regulated genes. In our view, a more informative way to choose *α *would be to let the user specify quantities such as average power or the false nondiscovery rate (FNR). We now discuss how this can be accomplished.

Power is defined as the probability of rejecting a hypothesis that is false. In the microarray multiple hypothesis setting, average power controls the proportion of regulated genes that is correctly identified. Setting *α *through an intuitively appealing measure as average power would thus be helpful. A relationship between *α *and average power can be found by rewriting the denominator of the right side in (13) as

Pr(*p *<*α*) = Pr(*p *<*α*|H_0 _true)*π*_0 _+ Pr(*p *<*α*|H_0 _false)(1 - *π*_0_).

Recognizing Pr(*p *<*α*|H_0 _false) as average power, (13) can be inverted to find

(15) $$\alpha =\frac{\text{pFDR}}{1-\text{pFDR}}\frac{1-\pi_{0}}{\pi_{0}}\cdot \text{average power}.$$

Combining (15) and (13) one can now find sample sizes that achieve a specified pFDR and average power, with no need of specifying *α*.

Another interesting measure to control is the false nondiscovery rate (FNR), the expected proportion of false negatives among the hypotheses *not *rejected. In other words, the FNR controls the proportion of regulated genes erroneously accepted as unregulated. We use a version of the FNR discussed in [15] called the pFNR = E(*A*_1_/*M*_*A*_|*M*_*A *_> 0), which, under the same assumptions as for the pFDR, can be stated as

pFNR = Pr(H_0 _false|H_0 _accepted).

Rewriting this probability in terms of pFDR and *α *yields

(16) $$\alpha =\frac{1-\text{pFDR}-\pi_{0}}{1-\text{pFNR}-\text{pFDR}}.$$

Again, specifying a pFNR will correspond to a specific choice of *α*. The pFNR approach to setting *α *could be interesting to use. One should note, however, that in the microarray setting this measure can sometimes be hard to apply. The reason is the potentially large *M*_*A*_, due to a high proportion of unregulated genes. A large *M*_*A *_makes pFNR numerically small, and a reasonable size may be hard to set.

Having chosen a pFDR and *α *(from average power or pFNR), we need to find a sample size that solves (13). To do this, we express Pr(*p *<*α*) via our mixture model (3) as

(17) $$\mathrm{Pr}(p<\alpha )=\int_{y>|y_{0}|}f(y;h)dy,$$

where *y*_0 _is the t-score corresponding to a p-value cutoff *α *for testing the null hypothesis. Since *f*(*y*) is a weighted sum of *t*_*ν *_(*δ*)s, the integrals needed to be taken are simply quantiles of (noncentral) t-distributions. Sample size affects (17) because all *t*_*ν *_(*δ*)s to be integrated are on the form $t_{s_{1}(n)}(\xi_{i}\sigma_{i}^{-1}s_{2}(n))$, with *s*_1_(*n*) and *s*_2_(*n*) dependent on *n*_1 _and *n*_2_. (Refer to pdfs of (1) and (2)). If the effect sizes ${\left\{\xi_{i}\sigma_{i}^{-1}\right\}}_{i=0}^{g}$ and the weights ${\left\{\pi_{i}\right\}}_{i=0}^{g}$ were known we could evaluate (17) for all *s*_1_(*n*), *s*_2_(*n*).

These parameters can, however, be found from the fitted model. To obtain the effect sizes we can equate $\delta_{i}=\xi_{i}\sigma_{i}^{-1}s_{2}(\tilde{n}),i=(0,...,g)$, where *ñ *is the sample size used in the fit. The weights are estimated directly. Having expressed Pr(*p *<*α*) in terms of sample size we aim to find one that solves (13). This problem can be reformulated as finding the root of the function

$$S(n)=\text{pFDR}\sum_{i=0}^{g}\left(\pi_{i}\int_{y>|y_{0}|}t_{s_{1}(n)}(\xi_{i}\sigma_{i}^{-1}s_{2}(n))dy\right)-\pi_{0}\alpha .$$

For finding the root of *S*(*n*) we implement a bisection method.

### Testing

To evaluate our approach we implemented the EM-algorithm and the sample size estimation method described above. We then ran tests on simulated data sets. For reasons discussed above we focused on controlling average power, along with the pFDR, in our sample size estimates.

When using simulated data sets it is possible to calculate the true sample size needed to achieve a given combination of pFDR and average power. To evaluate the performance of our method we compared our estimates to the true values. For comparison with existing approaches we also included the estimates made by the methods of Hu *et al*. [13], Pounds and Cheng [14] and Pawitan *et al*. [16].

#### Use of existing methods

In their paper Hu *et al*. discuss three different mixture models. In our comparison we used their truncated normal model, as this seemed to be the favored one. To produce sample size estimates using this model one needs to input the wanted pFDR and E(*M*_*R*_). As we wished to control pFDR along with average power, we calculated the E(*M*_*R*_) corresponding to each choice of pFDR and average power as E(*M*_*R*_) = average power·*m*(1 - *π*_0_)/(1 – pFDR). All tests were run with default parameters as found in the source code. In the implementation of Hu *et al*., however, three parameters (diffthres, iternum, gridsize) were missing default values. Reasonable choices (0.01,10,100) were kindly provided by the authors.

For the method of Pounds and Cheng one needs to input quantities called anticipated false discovery ratio (aFDR) and anticipated average power. For the estimates presented here we used the corresponding pFDR and average power combination. As Pounds and Cheng work with F-statistics, the t-statistics calculated in our tests were transformed accordingly. (If T follows a *t*_*ν *_distribution, then T^2 ^is distributed as *F*_1,*ν*_). In their implementation Pounds and Cheng set an upper limit, nmax, on the sample size estimates, and replace all estimates above nmax with nmax itself. The default value of nmax = 50 was replaced with nmax = 1000 in our tests. The reason for this was that sample size estimates of more than 50 could, and did, occur in the tests. Using a low nmax would then affect the comparison to the other methods. Apart from nmax all tests were run with default parameters as found in the source code.

In [16] Pawitan *et al*. discuss a method for fitting a mixture model to t-statistics. The fitted model is used to make an estimate of the proportion of unregulated genes, *π*_0_. The use of their method for sample size estimation is mentioned, but is not further explored or tested. The only input needed to fit a model is the number of mixture components. In our tests, this number was determined using the AIC, as suggested by Pawitan *et al*. in their paper. In the implementation of Pawitan *et al*. the assignment of non-central components close to the central one is not restricted. A preliminary test run using their unadjusted model fit showed that sample size estimates were greatly deteriorated by this. In our tests we therefore adjusted their fitted model by collapsing all non-central components within a given threshold (|*δ*| < 0.75) into the central one. Our model adjustment corresponds to the *π*_0_-estimation procedure used in the implementation of Pawitan *et al*. For our test runs we used our procedures to produce sample size estimates based on the models fitted by this method.

#### Test procedure and results

For the test results presented here we used *m *= 10000 genes, and *n*_1 _= *n*_2 _= 5 measurements per group. For the proportion of unregulated genes we examined the cases of *π*_0 _= 0.7 and *π*_0 _= 0.9.

In a first set of tests we considered sample size estimates in the case of normally distributed measurements and equal variances. In this setting we simulated data with and without a correlation structure. The true distribution of noncentrality parameters for the *m*_1 _= (1 - *π*_0_)*m *regulated genes was generated in the following way: A random sample was drawn from a *N*(1,0.5^2^) and a *N*(-1,0.5^2^) distribution. Both samples were of size *m*_1_/2, and together they made up the ${\left\{\xi_{k}\right\}}_{k=1}^{m_{1}}$ for the regulated genes in data set. The measurement variances were set to $\sigma^{2}=\sigma_{1}^{2}=\sigma_{2}^{2}=0.5^{2}$. The noncentrality parameters of the regulated genes were then calculated as ${\left\{\xi_{k}\sigma^{-1}\sqrt{n_{1}n_{2}{\left(n_{1}+n_{2}\right)}^{-1}}\right\}}_{k=1}^{m_{1}}$ (Refer to discussion of (1)). Based on our experience with microarray data analysis the above choices seem plausible for log-transformed microarray measurements. Since the true noncentrality parameters are all known, the true sample size needed to achieve a particular pFDR and average power can be calculated.

Correlation was introduced using a block correlation structure with block size 50. The reasoning behind such a structure was discussed in [27]. All genes, regulated and unregulated, were randomly assigned to their blocks. A correlation matrix for each group was then generated by first sampling random values from a uniform *U*(-1, 1) distribution into the off-diagonal elements of a symmetric matrix. Then, using the iteration procedure described in [28], we iterated to find the positive semidefinite matrix with unit diagonal that was closest to our randomly generated one.

Using the above approach we simulated data. For each test case we generated 50 data sets and made sample size estimates based on these. In an initial test run we wanted to evaluate our choice of using a larger number of mixture components, *g*, than what is suggested by the AIC or BIC criterion. To do so, two models were fitted to each simulated data set using our algorithm. One model had our chosen number of mixture components, the other had the number indicated by the AIC. Sample size estimates were then produced for both models. The reason for comparing with the AIC instead of the BIC is that the BIC, in this setting, will favor even fewer components than the AIC. In this initial run only uncorrelated data were used. The sample size estimation results are listed in Table 2. The average number of components chosen by the AIC and our method were, respectively, 6.1 and 11.8 for *π*_0 _= 0.7 and 5.0 and 10.1 for *π*_0 _= 0.9. Based on our findings we concluded that there may be an advantage to using more components than suggested by the AIC, and we used this larger number of components in the remaining tests. We then turned our attention to comparing the different approaches to sample size estimation. Uncorrelated and correlated data were generated and sample size estimates were produced using all four methods. The results are found in the upper half of Table 3. In general it seems that our estimates are close to their true values. Results are slightly better when there is no correlation between genes. As was to be expected, accuracy decreases, and standard deviation increases, with increasing power. This is probably related to the difficult problem of describing the distribution of noncentrality parameters well near the point of no regulation, i.e. close to *δ *= 0. The estimates of Hu *et al*. seem largely to be further from the true value than our estimates, and to be more conservative, but have lower standard deviation. The deviation from the true value is particularly high in estimates for high power. The estimates of Pounds and Cheng seem to deviate from the true value, be more conservative than our estimates and have higher standard deviation. The conservativeness of the estimates of Pounds and Cheng is seen from their own numerical tests as well, in which the estimated actual power exceeds the desired power. The estimates of Pawitan *et al*. appear to be better than those of Hu *et al*. and Pounds and Cheng, but still seem to be further from the true value than our estimates, and to have higher standard deviation. For high power there is a tendency of underestimating the needed sample size using this method.

**Table 2:** Evaluating the number of mixture components.

*π*_0_	pFDR	power	True	JMB (sd)	AIC (sd)
0.7	0.05	0.6	6	6 (0.3)	7 (0.5)
0.7	0.05	0.7	8	8 (0.5)	8 (0.8)
0.7	0.05	0.8	11	10 (1.1)	15 (4.0)
0.7	0.05	0.9	24	22 (2.5)	52 (22.5)
0.7	0.01	0.6	9	9 (0.7)	9 (0.5)
0.7	0.01	0.7	11	11 (0.8)	12 (1.0)
0.7	0.01	0.8	16	15 (1.8)	25 (8.4)
0.7	0.01	0.9	35	35 (3.6)	79 (34.4)
0.9	0.05	0.6	9	8 (1.1)	11 (3.8)
0.9	0.05	0.7	11	11 (2.4)	15 (5.6)
0.9	0.05	0.8	16	15 (4.1)	21 (8.5)
0.9	0.05	0.9	35	24 (7.9)	33 (13.7)
0.9	0.01	0.6	11	11 (1.9)	15 (6.3)
0.9	0.01	0.7	14	14 (3.2)	21 (8.8)
0.9	0.01	0.8	21	20 (6.3)	29 (12.7)
0.9	0.01	0.9	45	32 (11.1)	44 (21.8)

True and estimated per group sample sizes for simulated data sets having *π*_0 _= 0.7 and *π*_0 _= 0.9, and for different pFDR and average power cutoffs. The reported sample size estimate is the average of 50 such estimates rounded off to the nearest integer. The standard deviation (sd) was based on the corresponding 50 data sets. For each data set the estimation method introduced in this paper was used with two different choices for *g*, the number of mixture components. The JMB column (from the author names) lists the result using a *g *as discussed in this paper. The AIC column lists the results using the AIC criterion for choosing *g*.

Tests with normally distributed measurements were also run, in which the ${\left\{\xi_{k}\right\}}_{k=1}^{m_{1}}$ were drawn from gamma distributions and where variances differed according to the model discussed below. Results were similar to those discussed above (not shown).

In a second set of tests we wanted to simulate data from a model having the characteristics of a true microarray experiment. We also wanted to see how sample size estimates were affected if the assumptions of normality and equal variances did not hold. To accomplish this we based our simulation on the Swirl data set, which is included in the limma software package [29], and on a model for gene expression level measurements discussed by Rocke and Durbin [30]. The model of Rocke and Durbin states that

(18) *w *= *α *+ *μe*^*η *^+ *∈*,

where *w *is the intensity measurement, *μ *is the expression level, *α *is the background level and *∈ *and *η *are error terms distributed as *N *(0, $\sigma_{\varepsilon }^{2}$) and *N *(0, $\sigma_{\eta }^{2}$) respectively. Using the estimation method discussed in their paper we estimated the parameters of (18) for the Swirl data set. The estimated parameters $\left(\hat{\alpha },{\hat{\sigma }}_{\varepsilon },{\hat{\sigma }}_{\eta }\right)$ were, for the mutant: (394.12, 150.84, 0.18), and for the wild-type: (612.99, 291.40, 0.19). To generate a set of log-ratios representative of the same data we performed a significance analysis as outlined in the limma user's guide. Using a cutoff level of 0.10 for the FDR-adjusted p-values, we obtained a set of 280 log-ratios for genes likely to be regulated. Log-ratios for the regulated genes in our tests were sampled from this set with replacement. The true expression levels were generated by sampling from the background-corrected mean intensities of the genes in the mutant data set. To simulate microarray data for two conditions the following procedure was used: A set of log-ratios, and the true expression levels for one condition, were sampled. The true expression levels for the other condition were then calculated. Using the above-mentioned model, with their respective sets of parameters, measurements were simulated for both conditions and then log-transformed. To introduce correlation in this setting we added a random effect, *γ*, to the log-transformed measurements for each correlated block of genes. The *γ *was drawn from a *N *(0, $0.5\sigma_{\eta }^{2}$) distribution. The block size was again assumed to be 50, and the genes were assigned randomly to each block. The true sample size requirements were in this case estimated by repeatedly drawing data sets from the given model and calculating their average power and FDR on a fine grid of cutoff values for the t-statistics. A direct calculation is possible since the regulated genes are known.

After generating a model as described above we again simulated data with and without a correlation structure. For each test setting we sampled 50 data sets and made sample size estimates from the data using all four methods. The results are summarized in the lower half of Table 3. For the methods of Hu *et al*. and Pounds and Cheng the trend is the same as in the first set of tests. Our method seems to slightly overestimate the needed sample size, while the method of Pawitan *et al*. now interestingly provides the estimates closest to the true value. The standard deviations for the estimates of Pawitan *et al*. are still somewhat higher than ours.

**Table 3:** Evaluating sample size estimates from different methods.

			No correlation					With correlation				
*π*_0_	pFDR	power	True	JMB (sd)	HZW (sd)	PC (sd)	PMMP (sd)	True	JMB (sd)	HZW (sd)	PC (sd)	PMMP (sd)
0.7	0.05	0.6	6	6 (0.4)	11 (0.0)	11 (1.6)	6 (0.4)	6	6 (0.5)	11 (0.0)	11 (1.4)	6 (0.5)
0.7	0.05	0.7	8	8 (0.6)	18 (0.2)	14 (2.8)	7 (1.0)	8	8 (1.0)	18 (0.0)	14 (2.3)	7 (0.9)
0.7	0.05	0.8	11	11 (1.5)	39 (0.7)	20 (4.8)	9 (2.5)	11	11 (2.0)	38 (0.5)	19 (4.1)	9 (2.0)
0.7	0.05	0.9	23	24 (3.4)	146 (2.4)	30 (9.5)	13 (6.5)	23	23 (4.5)	145 (1.6)	29 (7.9)	15 (6.8)
0.7	0.01	0.6	9	9 (0.5)	17 (0.5)	16 (2.7)	9 (0.9)	9	9 (0.7)	17 (0.5)	16 (2.4)	8 (0.8)
0.7	0.01	0.7	11	11 (1.1)	28 (0.5)	21 (4.6)	10 (1.7)	11	11 (1.7)	28 (0.5)	21 (3.8)	10 (1.7)
0.7	0.01	0.8	16	16 (2.4)	60 (1.1)	28 (7.9)	13 (4.5)	16	16 (3.3)	60 (0.8)	27 (6.4)	13 (3.4)
0.7	0.01	0.9	34	37 (6.0)	231 (4.2)	42 (14.5)	18 (10.0)	32	36 (7.5)	229 (2.8)	40 (11.9)	22 (11.3)
0.9	0.05	0.6	9	9 (2.1)	16 (0.5)	24 (7.8)	9 (3.9)	8	9 (2.6)	16 (0.5)	23 (6.6)	9 (3.1)
0.9	0.05	0.7	11	11 (3.5)	27 (0.8)	31 (11.4)	11 (7.1)	10	12 (4.1)	27 (0.8)	30 (9.7)	11 (6.6)
0.9	0.05	0.8	16	16 (5.2)	59 (1.7)	41 (16.7)	15 (11.1)	14	16 (5.9)	58 (1.7)	41 (14.4)	15 (11.8)
0.9	0.05	0.9	34	26 (7.6)	227(6.6)	60 (26.8)	21 (17.9)	29	25 (8.5)	225 (6.7)	59 (23.1)	22 (18.8)
0.9	0.01	0.6	11	12 (3.3)	22 (0.7)	33 (11.8)	12 (6.0)	11	12 (4.1)	22 (0.6)	32 (9.9)	12 (4.9)
0.9	0.01	0.7	14	15 (5.3)	38 (1.2)	43 (16.9)	15 (10.4)	13	16 (6.0)	37 (1.2)	42 (14.4)	15 (9.9)
0.9	0.01	0.8	21	21 (7.4)	82 (2.6)	56 (24.3)	20 (15.6)	19	21 (8.4)	81 (2.7)	55 (20.9)	21 (16.8)
0.9	0.01	0.9	46	35 (10.0)	318 (10.0)	79 (37.2)	27 (24.3)	38	33 (11.3)	316 (11.3)	78 (32.0)	29 (25.1)
												
0.7	0.05	0.6	6	6 (0.2)	6 (0.0)	10 (1.0)	6 (1.1)	6	6 (0.3)	6 (0.0)	10 (1.1)	5 (0.7)
0.7	0.05	0.7	7	8 (0.7)	8 (0.3)	12 (1.7)	8 (2.4)	7	8 (0.7)	8 (0.1)	12 (1.8)	7 (1.6)
0.7	0.05	0.8	9	11 (1.4)	16 (0.4)	16 (2.9)	10 (4.9)	9	11 (1.5)	16 (0.2)	16 (3.2)	10 (3.8)
0.7	0.05	0.9	14	23 (4.1)	56 (1.2)	24 (5.5)	18 (11.3)	15	24 (5.3)	56 (1.0)	25 (6.1)	14 (7.8)
0.7	0.01	0.6	8	8 (0.6)	8 (0.0)	11 (1.7)	8 (2.2)	8	8 (0.7)	8 (0.0)	14 (1.8)	8 (1.0)
0.7	0.01	0.7	10	11 (1.1)	12 0.1)	14 (2.8)	11 (4.3)	10	11 (1.3)	12 (0.1)	18 (2.8)	10 (3.1)
0.7	0.01	0.8	13	16 (2.5)	23 (0.5)	24 (4.6)	15 (8.4)	15	16 (2.5)	23 (0.3)	24 (5.0)	13 (6.4)
0.7	0.01	0.9	26	36 (7.2)	84 (1.8)	35 (8.4)	27 (17.8)	30	37 (8.8)	83 (1.3)	34 (9.4)	20 (12.0)
0.9	0.05	0.6	8	10 (2.0)	7 (0.4)	21 (8.2)	8 (1.6)	8	10 (2.3)	8 (0.4)	24 (8.5)	9 (3.6)
0.9	0.05	0.7	9	13 (3.3)	11 (0.7)	27 (12.1)	10 (3.9)	9	13 (3.4)	12 (0.7)	32 (12.8)	11 (5.7)
0.9	0.05	0.8	12	18 (5.5)	21 (1.2)	37 (19.0)	13 (8.0)	13	19 (5.0)	23 (1.5)	44 (19.7)	14 (8.7)
0.9	0.05	0.9	24	31 (8.5)	76 (4.6)	54 (30.2)	18 (14.5)	25	31 (7.9)	85 5.4)	65 (32.6)	20 (14.5)
0.9	0.01	0.6	11	13 (3.1)	9 (0.5)	29 (12.6)	11 (2.4)	11	13 (3.6)	10 (0.6)	34 (13.2)	12 (5.6)
0.9	0.01	0.7	13	17 (5.0)	16 (0.6)	38 (18.5)	14 (6.2)	14	19 (5.0)	17 (0.8)	45 (19.6)	15 (8.2)
0.9	0.01	0.8	18	25 (8.1)	28 (1.5)	50 (27.2)	17 (11.8)	19	26 (7.1)	31 (1.9)	60 (29.2)	19 (12.3)
0.9	0.01	0.9	51	43 (11.4)	103 (6.2)	72 (42.8)	23 (19.8)	53	43 (10.9)	115 (7.9)	88 (46.3)	26 (19.7)

True and estimated per group sample sizes for simulated data sets having *π*_0 _= 0.7 and *π*_0 _= 0.9, and for different pFDR and average power cutoffs. The reported sample size estimate is the average of 50 such estimates rounded off to nearest integer. The standard deviation (sd) was based on the corresponding 50 data sets. Estimates made using the method discussed in this paper are termed JMB in the table (from the author names), while estimates made by the methods discussed by Hu *et al*. [13], Pounds and Cheng [14] and Pawitan *et al*. [16] are termed HZW, PC and PMMP respectively.

Note that, since the implementations of Hu *et al*. and Pounds and Cheng support only sample size estimates based on two-sample t-statistics (1), all tests listed are based on this statistic. Our implementation supports both types, and tests were also run to check the case of one-sample t-statistics (2). The results were similar to those of the two-sample t-statistics (not shown).

## Conclusion

We have in this article discussed a mixture model approach to estimating the distribution of noncentrality parameters, and thus effect sizes, among regulated genes in a two-sample comparative microarray study. The model can be fitted to t-statistics calculated from pilot data for the study. We have also illustrated how the model can be used to estimate sample sizes that control the pFDR along with other statistical measures like average power or pFNR. In the microarray setting our results will often also be approximately valid when using the FDR and FNR instead of the pFDR and pFNR. This is due to, referring to Table 1, that one frequently will have Pr(*M*_*R*_) ≈ 1 and Pr(*M*_*A*_) ≈ 1 in this setting. Sample size estimation methods like the one presented are useful in the planning of any large scale microarray experiment.

We examined the accuracy of our sample size estimates by performing a series of numerical studies. The conclusions are that our estimates are reasonably accurate, and have low variance, for moderate cutoffs in the error measures used. For stringent cutoffs we see a larger variance and a somewhat lowered accuracy. Overall our method seems to provide better results than the available sample size estimation methods of Hu *et al*. and Pounds and Cheng. We have also evaluated a method by Pawitan *et al*. for fitting a mixture model and its use in sample size estimation. The results using the method are good, and our tests suggest that optimizing the method of Pawitan *et al*. for use in sample size estimation could be interesting.

The decreased accuracy for stringent cutoffs found in the estimates is probably due to the difficult task of describing the distribution of regulated genes well near the point of no regulation, that is near *δ *= 0. It is important that the characterization of such genes is precise, but also that it does not affect the estimated distribution of the unregulated genes. Our solution in this article was to introduce a small neighbourhood around *δ *= 0 in which no other components are fitted. Better ways of differentiating between the two distributions close to 0 could be a subject of further study.

In our tests we also checked how correlation among the genes would affect the sample size estimates. We found that the estimates were only moderately affected. Nevertheless, we believe that estimation methods that incorporate correlation among genes is an important topic for future studies.

For the microarray setting the use of a moderated t-statistic, as discussed in [31], is often more appropriate than the ordinary t-statistic. For our method to be directly applicable to this type of statistic one would need to know that moderated t-statistics for unregulated genes follow a central t-distribution, and one would need the distribution's degrees of freedom. In [31] this distributional result is shown to hold, with augmented degrees of freedom for the central t-distribution. One would also need to know that moderated t-statistics for a regulated gene, with some given degree of regulation, follow a noncentral t-distribution, and one would need the distribution's degrees of freedom and noncentrality parameter. For this second result we are not aware of any findings. If this second result is shown to hold, then our method can be applied to moderated t-statistics as well. Testing the performance of our algorithm with moderated t-statistics, even without the second result, could also be interesting.

In our work we focus on average power as the control measure used along with the pFDR. One should note that having an average power of 0.9 means being able to correctly identify 90% of the regulated genes. In many experiments achieving this may not be interesting. One example is studies that aim only at identifying the small set of marker genes that best distinguishes one sample from the other. Other examples are experiments that, when comparing a treated and untreated tissue sample, only are interested in the most heavily affected regulatory pathways. In both these examples a power well below 0.9 could suffice. Our estimates are in this case particularly useful since they seem to be both accurate, and have low variance, for moderate power cutoffs with a low pFDR.

Although our main goal in this paper was fitting a model to be used in sample size estimation we would like to emphasize that the fitted model itself does provide some interesting information. One example is a direct estimate of *π*_0_, the proportion of unregulated genes, which we often found to be better than the one made by existing methods.

Other subjects for future work include a speed-up of the algorithm. The convergence of a straightforward EM-algorithm is known to be rather slow, and methods that improve on it will be implemented. Another issue that may be investigated further is picking the number of model components. As already mentioned, using a few more components than suggested by the traditionally used information criteria would often improve sample size estimates substantially. A different approach might be needed to choose the number of components in this setting.

The methods discussed in this article are applicable to any two-sample comparative multiple hypothesis testing situation where t-tests are used, and not only to problems in the microarray setting.

## Availability

An implementation of the methods discussed in this paper for the R environment is available from the authors upon request.

## Authors' contributions

HM and TSJ developed the statistical algorithms, AMB provided the biological insight. TSJ implemented the methods, ran the tests and drafted the manuscript. HM and AMB revised the manuscript. All authors read and approved the final manuscript.
